# Evidence for a Link Between *Fkbp5/FKBP5*, Early Life Social Relations and Alcohol Drinking in Young Adult Rats and Humans

**DOI:** 10.1007/s12035-016-0157-z

**Published:** 2016-10-05

**Authors:** Ingrid Nylander, Aniruddha Todkar, Linnea Granholm, Maria Vrettou, Megha Bendre, Wout Boon, Henrik Andershed, Catherine Tuvblad, Kent W Nilsson, Erika Comasco

**Affiliations:** 1 Department of Pharmaceutical Bioscience, Uppsala University, Box 591, SE-75124 Uppsala, Sweden; 2 Department of Neuroscience, Uppsala University, Box 593, SE-75124 Uppsala, Sweden; 3 School of Law, Psychology and Social Work, Örebro University, SE-70182 Örebro, Sweden; 4 Department of Psychology, University of Southern California, 3620 S. McClintock Ave, Los Angeles, CA 90089-1061 USA; 5 Centre for Clinical Research, Västerås Central Hospital, SE-72189 Västerås, Sweden

**Keywords:** Alcohol, Brain, Expression, FKBP5, Genotype, Stress

## Abstract

Alcohol misuse has been linked to dysregulation of stress, emotion, and reward brain circuitries. A candidate key mediator of this association is the FK506-binding protein (FKBP5), a negative regulator of the glucocorticoid receptor. The aim of the present study was to further understand the *Fkbp5/FKBP5*-related genetic underpinnings underlying the relationship between early life social relations and alcohol drinking. The effect of maternal separation and voluntary alcohol drinking on *Fkbp5* expression was investigated in the brain of young adult rats, whereas the interaction effect of the functional *FKBP5* single nucleotide polymorphism rs1360780 genotype and parent-child relationship on problematic drinking was examined in young adult humans. In rats, *Fkbp5* expression in the nucleus accumbens and ventral tegmental area, core regions of the reward system, was affected in a region-dependent manner and in opposite direction by maternal separation and alcohol drinking. *Fkbp5* expression in the cingulate cortex was affected by the combined effect of maternal separation and alcohol drinking. In humans, the TT genotype, in the presence of a poor relationship between the child and parents, was associated with problematic drinking behavior. The present findings suggest that *Fkbp5* expression in mesocorticolimbic dopaminergic regions associates with early life stress-mediated sensitivity to alcohol drinking and that *FKBP5* genotype interacts with parent-child relationship to influence alcohol drinking. These findings are the first to point to a role of *FKBP5* in propensity to alcohol misuse and call for studies of the underlying molecular mechanisms to identify potential drug targets.

**Electronic supplementary material:**

The online version of this article (doi:10.1007/s12035-016-0157-z) contains supplementary material, which is available to authorized users.

## Introduction

The FK506-binding protein 5 (FKBP5) has been recently proposed as a drug target for treatment of stress-related disorders [1, 2]. FKBP5, a co-chaperone of the heat shock protein 90 (Hsp90), can decrease the affinity for glucocorticoids [3, 4] by binding through a tetratricopeptide repeat protein domain on the premature Hsp90—glucocorticoid receptors (GR) complex and change its conformation [4, 5]. FKBP5 also promotes the internalization of the immature GR complex, thus reducing the GR complex’s activity as a transcription factor [6, 7]. Glucocorticoids, released from the adrenals as a response to a stressor, exert their actions through GRs widespread in the HPA axis, mesocorticolimbic and striatal circuitries. Overlapping expression patterns of *Fkbp5* with *Nr3c1* (the gene coding for GR) throughout the rodent’s brain [8], and its ability as a negative regulator of GR, render FKBP5 a putative target of studies on stress [9] and addiction.

Early life stress (ELS), in interaction with the genetic make-up, can alter programming of the brain during development [10]. ELS is suggested to interfere with the HPA axis development [11], having been associated with reduced GR expression, negative feedback regulation, and disrupted stress responsivity [12]. ELS, likely through malprogramming of the reward dopaminergic system [13], contributes to vulnerability to alcohol misuse [12, 14, 15]. The interplay between the hypothalamic-pituitary-adrenal (HPA) axis and the mesocorticolimbic and striatal systems is in fact critical for the stress response, emotion processing as well as alcohol seeking and intake [16, 17].

Corticosterone, the principal glucocorticoid in rodents, through GRs, increases dopaminergic activity in mesocorticolimbic areas, and reduced GR sensitivity is suggested to increase vulnerability to addiction [18]. Chronic corticosterone administration, which mimics chronic stress, leads to reorganization of the cortical neuronal circuits, likely compromising their top-down inhibitory function exerted on amygdala (Amy) activity [19, 20]. Under stressful conditions, greater Amy activity leads to increased dopamine levels in the nucleus accumbens (Acb) and medial prefrontal cortex (mPFC) possibly through stimulation of the ventral tegmental area (VTA) [18, 19].

Alcohol affects the mesocorticolimbic and striatal circuitries as well as the HPA axis response [21, 22]. The initial effect of alcohol is mediated by activation of dopaminergic projections from the VTA to the Acb [17]. The transition from habitual drinking to compulsive drinking involves the dorsal striatum (dStr), mediating the stress-induced seeking and habitual use of alcohol [13], as well as the cortical regions, such as the mPFC and cingulate cortex (CCx), which are responsible for cognitive control functioning [17]. Projections from the Amy to the HPA axis and Acb mediate the development of negative reinforcement [17]. Nevertheless, the molecular underpinnings of how ELS leads to alcohol misuse are still not well understood [23].

The *Fkbp5/FKBP5* genotype in rodents and humans, respectively, has been associated with differences in stress reactivity and alcohol withdrawal [9, 24–26]. A functional polymorphism in the human *FKBP5* gene has been identified, SNP rs1360780; homozygosity for the minor allele T has been associated with twice the amount of FKBP5 protein levels in lymphocytes, relative to the other genotypes [27]. Furthermore, differential stress responsivity, distinctive Amy overreactivity to emotional stimuli as well as psychiatric phenotypes characterized by an impaired stress response have been associated with the T allele or TT genotype, alone or in interaction with aversive environmental factors [9, 28].

Poor social interactions during early life, such as poor parent-offspring relationship, constitute a stressor than can influence proneness to alcohol misuse [10, 14]. To date, the relationship between mesocorticolimbic and striatal *Fkbp5* expression and early life stress-mediated propensity to alcohol drinking remains unknown. The present study investigated the effect of maternal separation and adult voluntary alcohol drinking on *Fkbp5* gene expression in the mesocorticolimbic and striatal areas of rats. In the presence of ELS, higher *Fkbp5* gene expression was expected in the Amy, alcohol was hypothesized to counterbalance the effect of ELS on *Fkbp5* gene expression in the Acb, whereas the direction of effects in other brain regions was not predicted. Furthermore, in a translational approach, the interaction effect between *FKBP5* rs1360780 genotype and parent-child relationship on problematic drinking was tested in a population-based sample of young adults. The hypothesis was that drinking problems would be more frequent among carriers of the T allele whom experienced a poor relationship with their parents.

## Methods and Materials

### Study of Rodents

The study was approved by the Uppsala Animal Ethical Committee (C32/11), according to the Swedish Legislation on Animal Experimentation (Animal Welfare Act SFS1998:56) and the European Communities Council Directive (86/609/EEC). The experiment is described in detail in the supplement and the study outline is presented as Fig. S1. Outbred Wistar male rats (RccHan:WI, Harlan, Europe) were used. Details and previous publications regarding this experiment are reported in the supplementary material.

#### Early Life Rearing Conditions

During the first three postnatal weeks, ELS was simulated by repeated prolonged maternal separations (MS) for 360 min (MS360), while brief MS with the same handling (15 min; MS15) was applied as control condition.

#### Voluntary Alcohol Consumption

On postnatal week 10, the MS rats were single housed and randomly assigned to water drinking (MS15W, *n* = 10; MS360W, *n* = 10) or free-choice water/alcohol drinking (MS15E, *n* = 10; MS360E, *n* = 20) groups. To simulate human episodic drinking, a voluntary drinking paradigm with repeated drinking and non-drinking days in between was employed [29]. The rats exposed to ethanol had free choice between non-sweetened ethanol (5 or 20 % made from Ethanol 96 %; Solveco AB, Rosersberg, Sweden) and water for three consecutive days a week with drug-free days in-between. The first week the rats had free access to 5 % ethanol for 24 h/day, the next week they had limited access to 5 % for 2 h/day, and the following five weeks access to 20 % ethanol in 2 h sessions for three consecutive days a week. Water drinking controls had two bottles with water. After 5 weeks of access to 20 % ethanol and/or water, the rats were decapitated immediately after a 2-h drinking session. Trunk blood was collected, and the brain regions VTA, Acb, Amy, dStr, mPFC, and CCx were removed, immediately frozen, and stored at −80 °C.

#### Genetic Analyses

RNA was isolated and converted to cDNA. Diluted cDNA (20×) was used to assess the expression of *Fkbp5* in Amy, Acb, mPFC VTA, dStr, and CCx using CFX96 Touch Real-Time PCR Detection System real-time PCR (BioRad, USA) (Table S1). Relative gene transcripts levels in all animals of each experimental group were determined using the ∆CT method (Biorad real time PCR application guide) (Table S2; Fig. S2 and S3).

#### Corticosterone Measurement

Samples were analyzed as described in [30].

### Study of Humans

The Regional Ethical Review Board approved the study (2010/463). Individuals were participants of the Retrospective Study of Young People’s Experiences (RESUME), a Swedish population-based, cross-sectional, and retrospective study. Only males were studied since only male rats were investigated, the cohort is described in the Supplement, (*N* = 838). The participants completed a questionnaire and provided a saliva sample and received a small monetary compensation [31]. Details and previous publications regarding this study are reported in the supplementary material.

#### The Alcohol Use Disorders Identification Test

All participants completed the 10-item Alcohol Use Disorders Identification Test (AUDIT) questionnaire regarding quantity (number of standard drinks = 12 g alcohol), frequency of drinking as well as alcohol-related problems. The results for each question (score range 0–4) were summed to obtain the AUDIT score (i.e., total score range 0–40) (Cronbach’s alpha = 0.752). Scoring above eight points in males has been suggested as index of problematic drinking [32].

#### Parent-Child Relationship

Parent-child relationship was measured using the following items: “When you were growing up (0—18 years), how often did your (1) mother, (2) father, show clearly that they liked you, for example, by asking it or giving you a hug or kiss?” These items had a four-point response format, ranging from “not very close at all” to “very close.” A parent-child relationship summation index (ranging from 0 to 6) was created with a higher score indicating a more positive parent-child relationship. When answering these questions, 98.4 and 95.2 % referred to the biological mother and father, respectively.

#### Genotyping

DNA was extracted from 200 μl of saliva collected with the Oragene self-collection kit (DNA Genotek®) using the silica-based Kleargene DNA extraction method. Genotyping analyses of the single nucleotide polymorphism (SNP) rs1360780 were performed using the Kbioscience Allele-Specific Polymorphism assay based on competitive allele-specific PCR and bi-allelic scoring. No-template control samples were included to enable the detection of contamination or non-specific amplification.

### Statistical Analyses

Between-group differences were tested using the Mann Whitney *U* test. The General Linear Model (GLM) test with type III sum of square was used to assess the interaction effect between ELS and drinking, partial eta-squared value were used as effect size index. Group-wise bivariate correlation tests were performed using the Spearman’s rank test, and Bonferroni correction for multiple testing was applied. The statistical power of the human study was computed using the Genetic Power Calculator [33]. The size of the population-based sample had >80 % of statistical power considering *α* = 0.05 and heritability = 1 %. The interaction effect of *FKBP5* rs1360780 genotype and parent-child relationship on AUDIT was estimated by univariate ANOVA GLM test with type III sum of square test, which allows adjusting for the main effect of the two factors of interest and is considered robust to violations of normality [34]. As confirmatory test, binomial regression was performed, adjusted for the main effect of genotype and environment. For illustrative purposes, fit lines were computed using standard linear regression with the least squares method. All analyses were performed using the Statistical Package for the Social Sciences software v. 21.

## Results

### Study of Fkbp5 Expression in Rodents

**Fig. 1:**
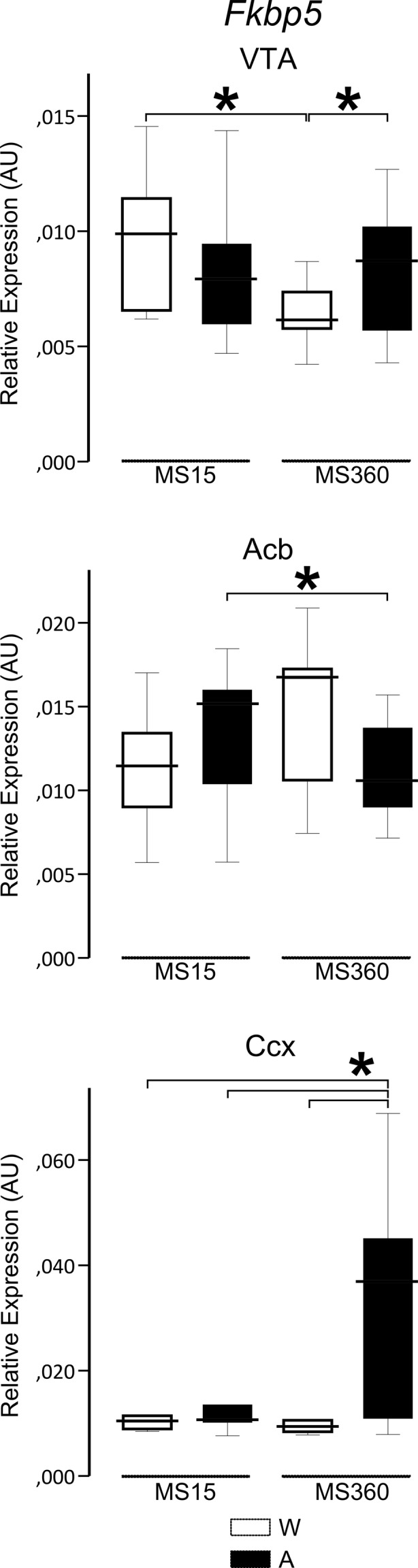
Median and IQ range of *Fkbp5* expression in MS rats drinking water or alcohol in a two-bottle free-choice model. *A* alcohol, *Acb* nucleus accumbens, *AU* arbitrary units, *CCx* cingulate cortex, *MS* maternal separation (15 or 360 min), *VTA* ventral tegmental area, *W* water; *p ≤ 0.05

In the VTA, water-drinking MS15 rats displayed higher *Fkbp5* expression compared to MS360 rats (*U* = 14, *p* = .005). Alcohol- and water-drinking MS15 rats did not differ in expression levels, whereas alcohol-drinking MS360 rats had higher *Fkbp5* expression than water-drinking MS360 rats (*U* = 48, *p* = .045) (Fig. 1). In the Acb, alcohol-drinking MS15 rats displayed higher expression of *Fkbp5* than alcohol-drinking MS360 rats (*U* = 52, *p* = .050) (Fig. 1). In the CCx, water-drinking MS15 rats (*U* = 49, *p* = .024), alcohol-drinking MS15 (*U* = 52, *p* = .035), as well as water-drinking MS360 rats (*U* = 38, *p* = .005) displayed lower *Fkbp5* expression compared to alcohol-drinking MS360 rats (Fig. 1). No group differences were observed on *Fkbp5* expression in the Amy, mPFC, and dStr (data not shown).

Interaction effects between ELS and ethanol drinking were observed in the VTA (*F* = 4.35, *p* = .043, ${\eta}_p^2$ = .090) and Acb (*F* = 5.16, *p* = .028, ${\eta}_p^2$ = .103) (Fig. S4), but not in other regions except a trend in the Ccx (*p* = 0.098) (data not shown). Regarding correlations of *Fkbp5* expression in different regions, a positive correlation was observed between mPFC and Amy (*r*_*s*_ = .740, *p* < .001) as well as between mPFC and CCx (*r*_*s*_ = .626, *p* = .003) in alcohol-drinking MS360, but not MS15, rats. Correlations that did not pass Bonferroni correction are reported in the Table S3 (non-significant correlations are not reported).

As reported in [30, 35], an interaction effect of ELS and ethanol drinking was found on corticosterone levels with alcohol-drinking MS360 rats displaying lower corticosterone levels compared to alcohol-drinking MS15 as well as water-drinking MS360 rats. A negative correlation was observed between *Fkbp5* expression in the mPFC and corticosterone levels in alcohol-drinking MS15 rats (*r*_*s*_ = −.943, *p* = .005) (Table S3).

Alcohol intake data have been previously reported [30, 36, 37]. The MS15 and MS360 groups did not differ in alcohol consumption although the propensity to increase alcohol intake over time and consume >1.5 g/kg/2 h was more evident in the MS360 group [37], as also reported in several studies [38]. Alcohol intake during the last week before sacrifice as well as the alcohol intake during the last week and last session was not correlated with *Fkbp5* expression in any brain region or group.

### Study of FKBP5 Genotype in Humans

**Fig. 2:**
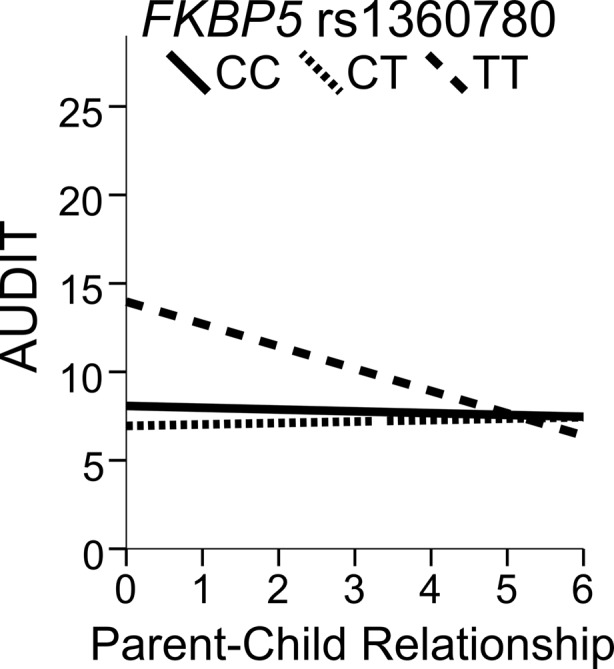
Fit regression lines between AUDIT score and parent-child relationship (0–18 years) by *FKBP5* genotype. A negative slope characterized TT carriers (*R* ^2^ = 0.11) whereas no association was present in C carriers

The mean age of the 838 young male adults was 22.1 ± 1.4 (20–24) years for which *FKBP5* rs1360780 genotype data were available (HWE *χ* ^2^ = 0.66, *p* = 0.42), and the age at first intoxication was 15.7 ± 1.9 years. The majority reported to drink 2 to 4 times a month, and consumed on average 5–6 drinks containing alcohol in a typical occasion, with AUDIT scores of 7.6 ± 5.2 (0–29). They reported that their relationship with their parents until the age of 18 years was 4.3 ± 1.5 on a scale between 0 and 6. Only 3.9 % never lived with both biological parents from birth to the age of 18, whereas 60.7 % always lived with both biological parents. Though the *FKBP5* rs1360780 TT genotype group associated with higher AUDIT scores, *FKBP5* rs1360780 genotype frequencies (CC: 53.2 %, TC: 40.2 %, TT: 6.6 %) did not differ regarding alcohol intake or relationship with the parents (Table S4). No correlation was observed between AUDIT and parent-child relationship, except among TT carriers for whom problematic drinking was associated with poor parent-child relationship (*r* = 0.353; *p* < 0.008). The *FKBP5* rs1360780 genotype modulated this association, with carriers homozygous for the T allele scoring the highest in AUDIT when experiencing a poor relationship with the parents (*F*_(2, 832)_ = 3.6; *p* = 0.027) (Fig. 2).

## Discussion

To examine the relation between *Fkbp5/FKBP5*, early life social interactions and alcohol drinking, a translational approach studying rodents and humans was used. The expression of the *Fkbp5* was assessed in the mesocorticolimbic and striatal areas of young adult outbred rats exposed to an experimental model of ELS and later to an episodic drinking paradigm. The present study for the first time provides mechanistic evidence of long-term effects of maternal separation and also different alcohol-induced effects depending on early life conditions, on *Fkbp5* expression in dopaminergic key regions of the reward system in outbred male rats. Moreover, the interaction effect between *FKBP5* rs1360780 genotype and parent-child relationship on problematic drinking was tested in a population-based sample of young male adults. The TT genotype, in interaction with poor parent-child relationship, was associated with problematic drinking. These findings expand our knowledge on the relations between alcohol drinking, early life social interactions, and *Fkbp5* expression in rodents as well as *FKBP5* genotype in humans.

## *Fkbp5* Expression in Relation to Maternal Separation and Alcohol Drinking in Rats

Knowledge on the biological underpinnings mediating the impact of ELS on alcohol misuse is scarce [22, 39]. Disrupted dam-pup interactions during the first postnatal weeks before weaning have been widely advocated as a maladaptive developmental pathway to unfavorable neurobiological and behavioral outcomes [23]. Maternal separation in early life and intermittent voluntary alcohol drinking in young adulthood, two experimental paradigms with high face and construct validity, were herein employed to explore their shaping influence on *Fkbp5* gene expression. To date, few studies investigated the relationship between *Fkbp5* and alcohol drinking and none the interaction with ELS. Intraperitoneal administration of alcohol was shown to increase *Fkbp5* expression in the murine striatum putamen and Acb [40], whereas higher withdrawal-like symptoms were displayed by *Fkbp5* knock-out compared to wild-type mice [26].

ELS associates with long-term molecular changes in the mesocorticolimbic and striatal systems increasing vulnerability to addiction [13]. Herein, the effect of ELS-mediated sensitivity to alcohol drinking on *Fkbp5* expression localized in the mesocorticolimbic, but not striatal, dopaminergic brain regions. An opposite effect of ELS and alcohol was observed on *Fkbp5* expression in the VTA and Acb. In the VTA, rats exposed to ELS through prolonged maternal separation (MS360) had lower *Fkbp5* expression than the MS15 rats. Furthermore, alcohol drinking affected *Fkbp5* expression only in the MS360 rats, which displayed higher levels, towards those seen in the MS15 rats. The gene expression difference in response to voluntary drinking could contribute to the higher propensity for excessive alcohol intake that usually emerges after 3 to 5 weeks of voluntary consumption in MS360 compared to MS15 rats [14]. Interestingly, in the VTA, the direction of the interaction effect was opposite to that in the Acb, likely indicating region-dependent molecular mechanisms involving *Fkbp5* in relation to ELS and alcohol in dopaminergic projecting compared to receiving areas, VTA vs. Acb.

In the present study, water-drinking rats exposed to ELS tendentially displayed the highest corticosterone levels [30], which is in line with the long-term impact of maternal separation and the hyporesponsivity of the HPA axis seen in rats exposed to high dam-pup interactions during early postnatal life [23]. Glucocorticoids released as a result of stress have been associated with neuroadaptations in the VTA through direct activation of GR [41], thus supporting a role of FKBP5. Persistent higher corticosterone levels follow prolonged MS in animal models of ELS [42] and are associated with decreased dopaminergic output in the Acb [43]. Since FKBP5 is a negative regulator of GR, the tendentially higher *Fkbp5* expression observed here in the Acb of water-drinking rats exposed to ELS might be part of a mechanism balancing their higher corticosterone levels. On the other hand, alcohol appeared to reverse these effects. Alcohol-drinking rats exposed to ELS compared to controls displayed lower *Fkbp5* expression in the Acb but higher *Fkbp5* expression in the VTA. Additionally, these rats had lower corticosterone levels [30], in line with the dampening of the stress response by alcohol observed in individuals at risk to develop alcohol use disorder [21].

While the interactive effect between ELS and alcohol seemed to normalize *Fkbp5* expression in the reward circuit to the levels observed in control rats, a unique effect was observed in the CCx. Alcohol-drinking rats exposed to ELS displayed higher *Fkbp5* expression compared to all other groups. The CCx regulates goal-oriented decision-making, outcomes representation and value [44], which are likely to be altered in the presence of ELS and alcohol. Altogether, these findings point to FKBP5 as a modulator of dopaminergic neurotransmission not only in processing of reward and motivational salience but also in executive functioning, supporting the dopamine hypothesis of addiction [45].

As key actors of the dopaminergic system, the VTA and Acb are involved in incentive salience to promote goal-directed behavior, whereas the latter is also part of the extended amygdala complex which integrates the stress-arousal systems with hedonic processing to produce emotional states [17]. Interaction effects could have also been expected in the Amy which is involved in emotion regulation and modulation of the reinforcing effects of alcohol [17]. However, no difference was observed in *Fkbp5* expression in the Amy and not in mPFC, another stress-regulating area. Higher *Fkbp5* expression has been observed upon restraint stress in the murine Amy as well as in hypothalamic and extra-hypothalamic regions in response to dexamethasone or corticosterone challenge [8, 46]. Knocking out of *Fkbp5* in the Amy or the entire brain has been linked to lower fear responses, corticosterone levels, and stress induced fear response, as well as shorter recovery time after chronic social defeat stress in mice [24, 25]. Analysis of the central nucleus of the amygdala, which mediates acute reinforcing actions, separately from the basolateral amygdala, which mediates processes conditioned reinforcing and drug-induced reinstatement [17], might have unveiled differential *Fkbp5* expression profiles in the present study. A positive fronto-amygdala correlation of Fkbp5 expression was present in alcohol-drinking rats exposed to ELS, suggesting an Fkbp5-mediated interplay between executive and emotional regulating areas under the interactive influence of ELS and alcohol. While the absence of correlations between the VTA, Acb, and Ccx could be interpreted as a consequence of the herein observed effects of maternal separation and alcohol which disrupted potential relationships between these regions. On the other hand, the lack of interaction effects on *Fkbp5* expression in the mPFC and dStr might be explained by the fact that the rats were sacrificed at a time point when habitual and compulsive stages of alcohol drinking, which are mainly mediated by cortical and striatal regions, were not yet well established. Finally, the strong negative relationship between corticosterone and gene expression in the mPFC of alcohol-drinking control rats and its absence in other groups calls for further investigation on the underlying mechanism.

## *FKBP5* Genotype in Relation to Parent-Child Relationship and Problematic Drinking in Humans

The quality of parent-child interactions can widely vary in humans, from emotional warmth and physical affection to neglect and detachment. This impacts attachment patterns and consequently the emotional and social development of the offspring [47]. Insecure bonding, such as childhood neglect, has been linked to early alcohol initiation and alcohol misuse [47–51]. Early environmental adversity can indeed program the stress response system and imprint brain development with long-term repercussions on neuroplasticity, behavior, and vulnerability to addiction [52]. Nevertheless, not every individual exposed to environmental stressors will misuse alcohol; yet an adverse environment is likely to be a prerequisite for the penetrance of genetic risk factors [15, 52]. Therefore, both constitutional and psychosocial factors are of importance to the risk trajectory that ultimately can culminate into addiction [15].

Signatures of parent-child relationship on both the reward and stress systems are left during development [53]. The association between alcohol misuse and disrupted early life social interactions between the parent and offspring is presumably driven, in part at least, by interactions with genes related to stress reactivity and emotion processing [16]. Parent-child relationship was here studied in relation to *FKBP5* genotype and problematic alcohol drinking in young male adults. The TT genotype, in the presence of a poor relationship between the child and the parents, was associated with problematic drinking.

The *FKBP5* gene variant examined in the present study has not only genetic [27] but also endocrine and neural functional effects, with the T allele being the vulnerability factor [9]. It is likely that insecurity and negative affect, derived from a poor relationship with the parents, are reasons for alcohol misuse in individuals carrying the more stress-sensitive *FKBP5* genotype. Indeed, T/TT carriers have been described to have heightened amygdala reactivity to negative emotional stimuli contingent to emotional neglect [54, 55]; increased activation during attention bias towards threat and spatial displacement of the hippocampus in the presence of trauma [56]; lower white matter integrity in the posterior cingulum [57] and in the dorsal anterior and posterior cingulate cortex [58], which connect emotional and cognitive processes; weakened connectivity at rest between left amygdala and caudate, parahippocampal gyrus, inferior and middle frontal gyri, in interaction with negative life events [59]; as well as differential gray matter volumes in brain regions involved in cognitive-affective processing [55, 58, 60], in some cases dependently on experience of adversities [61, 62].

This is to our knowledge the first attempt to investigate the relationship between *FKBP5* genotype and alcohol misuse in the context of parent-child relationship. Huang and colleagues demonstrated greater predisposition to develop fewer withdrawal symptoms in patients with alcohol use disorder carrying the T allele [26], a factor that can lead to greater alcohol misuse. More recently, the minor allele of a highly linked SNP, rs9296158, has been associated with reward dependent personality, worse working memory, and blunted autonomic response to stress, among young adults exposed to early life adversity [63]. Binder and colleagues elegantly proved the T allele to enhance *FKBP5* gene transcription by enabling the interaction between a glucocorticoid response element and the transcription start site, thus influencing the response to the glucocorticoid receptor activation triggered by early life adversity [64]. All together, these findings provide complementary evidence suggesting a heightened predisposition to stress- and emotion-driven alcohol misuse among individuals carrying the T/TT of the *FKBP5* rs1360780 SNP.

Addiction is a polygenetic and multifactorial phenotype [65], and several gene-environment association studies have been carried out in the last decades; however, mixed results have been found [66]. The present study, by using a relatively large sample, focused on a functional genetic correlate of stress sensitivity and emotion processing [9, 27]. Yet, the effect of a single genetic polymorphism typically accounts for a minor percentage of the variance in a phenotype [67], and contributes in concert with other factors to explain a certain phenotype, thus the study of stress-related polygenic risk indexes is prompted. Of relevance to the dopaminergic hypothesis of addiction, the interaction between attachment and dopaminergic genotype has been investigated in relation to alcohol use problems in young adults, but no association has been found [68]. Moreover, females should also be studied, since sex differences in stress reactivity and addiction are rather the rule than the exception [69, 70]; *FKBP5* genotype was for instance found to mediate the cortisol stress response in male but not female young adults [71]. The findings on humans have to be understood with respect to these boundaries (see also Strengths and Limitations) and call for independent replications.

## Strengths and Limitations

Experimental animal studies can provide insight into the molecular mechanisms underlying the impact of ELS on brain function and behavior [11, 22]; translational approaches can corroborate them. Similarly, to the human population-based sample, outbred rats were investigated. Episodic voluntary drinking was used to simulate human habitual drinking [29], therefore possessing high face validity. A consumption pattern with repeated drinking days and non-drinking days in-between is also known to associate with neurobiological changes similar to those seen in the transition from habitual to compulsive drinking [72]. Construct validity, as underlying biology, and predictive validity, as response to alcohol, are also likely to replicate human phenotypic endpoints. Despite differences in the environment (e.g., social stimuli), these characteristics support the translational interpretation of molecular underpinnings of individual variations in vulnerability to alcohol misuse.

Parent-child relationship was employed as proxy of early life interactions between caregiver and offspring; however, the psychometric property of these items is debatable. The attachment construct comprises, among others, bonding typology, parents’ responsiveness, and parenting style, which were not considered herein. Moreover, the present data are a representation of the parent-child relationship only from the offspring’s perspective. On the other hand, parental deprivation can only be assessed in samples of orphans, though institutional care can be seen as a form of parenting. Almost all individuals spent their first 18 years living with their biological parents, thus strengthening the homogeneity of the present sample and not allowing to investigate the effect of institutional care. Several unconventional typologies of rearing and caregiving are nowadays common (e.g., single-parent family, adoptive parents, homosexual parents, parents from patchwork families, parents via surrogacy), a factor related to attachment that should be investigated in future studies. Additionally, over the time, the parent-child relationship develops from sensitiveness to visual, tactile and auditory cues, to reciprocity and mutuality, into behavior guidance, parental regard, and independence. The present measure presumably reflects the combination of them, though likely biased by recent memories, since the participants were asked to recall their impression over a period of 18 years.

Notably, causative links could not be estimated in the present cross-sectional study, nonetheless bidirectional influences have also to be expected. Attachment, besides being a predictor of behavior during adulthood and alcohol misuse, has also been shown to be negatively affected by alcohol misuse [51]. Nevertheless, the study of rats points to FKBP5-mediated long-term effects of ELS and its interaction with alcohol consumption. The time point when the rats were sacrificed coincides with the beginning of the establishment of individual alcohol consumption patterns and the beginning of transition into high alcohol intake in the MS360 rats [38]. That is, the rats are not in the addictive state and large differences in alcohol exposure do not confound the results. This serves the purpose to investigate early neurobiological signatures related to the propensity to escalate into excessive drinking. Likewise, the young adults did not yet fulfill the criteria for a diagnosis of AUD (only 2 % scored above 19 on the AUDIT, the threshold for requiring referral to specialist for diagnostic evaluation and treatment); this allowed studying the early stage of the trajectory to addiction. It is indeed of utmost importance to consider early life factors and the period of young adulthood, which is a key developmental period of the brain accompanied by behavioral traits such as risk taking and novelty seeking, and psychosocial factors such as drinking for coping reasons that can predispose to alcohol misuse.

Last but not least, the functionality of the investigated polymorphism, which has been proven not only in terms of protein levels but also HPA axis response, aims to overcome the fact that *Fkbp5* gene expression in vivo cannot be assessed in the human brain. On the other hand, rodents do not carry an orthologous polymorphism. This is therefore an imperfect translational study whose results can be difficult to interconnect and should be seen as a valuable complement to each other.

## Conclusion

The present findings are the first to point to a role of *Fkbp5/FKBP5* in the association between early life social interactions and alcohol consumption in both rodents and humans. The mesocorticolimbic, and not striatal, circuit was sensitive to the dual effects of maternal separation and alcohol mediated by *Fkbp5* in rats. *FKBP5* genotype modulated the association between parent-child relationship and alcohol misuse in young adults. Future studies should explore *Fkbp5*-related molecular mechanisms in dopaminergic projecting and receiving areas to advance understanding of the effect of ELS on vulnerability to addiction, causal relationships as well as the effects of drugs targeting FKBP5.

## Electronic Supplementary Material

ESM 1 (DOCX 2235 kb)
