# Supplementary material for: Evidence for a Link Between Fkbp5/FKBP5, Early Life Social Relations and Alcohol Drinking in Young Adult Rats and Humans
Source: Mol Neurobiol. 2016 Oct 5;54(8):6225–34. doi: 10.1007/s12035-016-0157-z (PMC5583263; doi:10.1007/s12035-016-0157-z)
Supplement: Supplementary file 1 — (DOCX 2235 kb) [file 12035_2016_157_MOESM1_ESM.docx]

Supplementary materials and methods

**Evidence for a link between *Fkbp5/FKBP5,* early life social relations and alcohol drinking in young adult rats and humans**

Ingrid Nylander, Aniruddha Todkar, Linnea Granholm, Maria Vrettou, Megha Bendre, Wout Boon, Henrik Andershed, Catherine Tuvblad, Kent W Nilsson, Erika Comasco

**Materials and Methods**

## *Ethical Permission*

The experimental study of rodents was approved by the Uppsala Animal Ethical Committee (C32/11) and followed the guidelines of the Swedish Legislation on Animal Experimentation (Animal Welfare Act SFS1998:56) and the European Communities Council Directive (86/609/EEC). The study of humans was approved by the Regional Ethics Review Board (#2010/463).

## *Experimental Study of Rodents*

Time mated Wistar dams (n = 25; RccHan:WI, Harlan, Europe) arrived at gestation day 15. After birth (postnatal day (PND) 0) the pups were sexed and cross-fostered to avoid the use of biological littermates in the same experimental groups. Each litter contained 10 pups, 6 males and 4 females, and the litters were randomly assigned to the different experimental groups. Rats used in the present study were only males and their brain has been previously used to address other research questions [1-4].

*Early Life Rearing Conditions*

Based on previous studies, prolonged daily maternal separation (MS) for 360 min (MS360) was used as a risk environment for first three postnatal weeks (Figure S1), and short MS (15 min; MS15) as control to MS360 [5]. MS15 and MS360 rats were exposed to the same handling procedures during MS with only the length of the separation being different. The separations were performed during the light period and started at 9 AM. The MS procedure has been described in detail elsewhere [6]. The litters were weighed on PND 0, 3, 7, 10, 13, 16 and the cages were changed on PND 7 and 16. The separations were always performed in the same animal rooms and only one person performed all separation and care taking. On PND 22 all animals were weaned, the light/dark-cycle was switched to lights off between 06.00 and 18.00 until the end of the experiment, and then group-housed three per cage, during adolescence.

*Voluntary Ethanol Consumption*

On postnatal week 10, the MS rats were single housed and randomly assigned to water-drinking (MS15W, n = 10; MS360W, n = 10) or ethanol drinking groups (MS15E, n = 10; MS360E, n = 20). More rats were included in the MS360 group based on previous findings of subgroups with responder and non-responder rats regarding effect on ethanol intake [5].

The rats exposed to ethanol had free choice between non-sweetened ethanol (5% or 20% made from Ethanol 96%; Solveco AB, Rosersberg, Sweden) and water for three consecutive days a week with drug-free days in-between. The first week the rats had free access to 5% ethanol for 24 h/day, the next week they had limited access to 5% for 2 h/day, and the following five weeks access to 20% ethanol in 2 h sessions for three consecutive days a week. This drinking paradigm is developed to mimic human episodic drinking patterns, with repeated drinking days and non-drinking days in between [7]. Water drinking controls had two bottles with water. Ethanol and water were changed every session and the bottle position was altered every day to avoid position preference. Bottles with nipples were employed to minimize spillage. At the end of each session, the ethanol and water intake was quantified by weighing the bottles. After 5 weeks of access to 20% ethanol, the rats were decapitated. The ethanol drinking animals were sacrificed immediately after a 2 h drinking session. Trunk blood was collected, and the brain regions VTA, Acb, Amy, dStr, mPFC, and CCx were removed, immediately frozen on dry ice, and stored at -80°C.

*Genetic Analyses*

RNA was isolated from the brain regions of interest using AllPrep DNA/RNA/miRNA Universal Kit according to the manufacturer’s protocol (Qiagen AB Sollentuna, Sweden). Quantification of RNA was carried out using a Nanodrop ND 1000 spectrometer. Each plate contained samples belonging to all experimental groups. Also, one reference sample was included in each plate for the same gene and later used for controlling plate bias.

*Gene Expression Analyses*

cDNA synthesis: RNA (700ng) was converted to cDNA using QuantiTect Reverse: Transcription Kit (Qiagen AB Sollentuna, Sweden). The manufacturer’s protocol was followed including a genomic DNA (gDNA) wipe-out reaction. The final cDNA synthesis reaction was carried out at 42°C for 35 minutes. The reaction was inactivated at 95°C for 5 minutes. Synthesized cDNA was diluted 20x and stored at -20°C until further use. gDNA contamination was controlled at three check-points: 1) on column DNase I treatment during the extraction process; 2) gDNA wipe-out reaction prior to cDNA synthesis; and 3) designing of primers across two adjacent exons to avoid any unspecific amplification of genomic DNA. Diluted cDNA (20x) was used to assess the expression of *Fkbp5* in Acb, Amy, CCx, dStr, mPFC and VTA using CFX96 Touch Real-Time PCR Detection System real time PCR. Primers were designed using Primer 3 (http://frodo.wi.mit.edu/) and cross-checked using Primer Map (http://www.bioinformatics.org/sms2/primer_map.html). For each sample, a 20µl reaction contained 1x [**iQ SYBR Green Supermix**](http://www.bio-rad.com/en-se/sku/170-8882-iq-sybr-green-supermix) (Biorad Sweden), 0.15µM of each primer, and 3 µl cDNA template. The PCR conditions consisted of denaturation at 95°C for 3 minutes followed by a three step protocol with denaturation at 95°C, annealing at the optimum temperature (see supplementary table 1), and elongation at 72°C. The single plate read was retrieved at the end of each elongation step, and the melt curve value was added after the end of the PCR from 65°C to 95°C with 0.5°C/cycle increments. Each rat cDNA and controls were run in triplicates. Data analysis: Data of the relative fluorescence units (RFU) were collected. PCR efficiency and Cq values were calculated using the LinregPCR (version 2015.3) (http://www.hartfaalcentrum.nl/index.php?main=files&sub=0) [8]. Using the reference control samples of each plate for the same gene, Cq values were further corrected for plate bias. Samples with Cq values above 0.4 standard deviations were excluded. Mean values of three housekeeping genes for each sample were used to calculate relative gene expression, except for the dorsal striatum where *Gapdh* displayed an effect of grouping and hence was excluded, and for the ventral tegmental area where the *Actb* PCR was not successful. Relative gene transcripts levels were determined using the ∆CT method (Biorad real time PCR application guide). All the laboratory and pre-processing analyses were performed in a blind manner.

*Corticosterone Measurement*

Samples were analyzed, as previously reported [1], using the commercial ImmuChemTM Double Antibody Corticosterone ^125^ I RIA kit for rats and mice (MP Biomedicals LLC, Orangeburg, NY, USA) in accordance with the included protocol, with the exception of the addition of one standard (12.5 ng/ml). All samples were analyzed in duplicate. According to the protocol of the RIA kit, the intra-assay variation was 4.4 – 10.3% and the inter-assay variation 6.5 – 7.2%. The corticosterone antiserum showed 100% cross-reactivity with corticosterone, while cross-reactivity to other steroids was 0.34% to deoxycorticosterone, 0.10% to testosterone, 0.05% to cortisol, and < 0.05% to other tested steroids.

***Study of Humans***

Participants in the present study were drawn from the Retrospective Study of Young People’s Experiences (RESUME), which is a Swedish population-based, cross-sectional and retrospective study [9]. As described in [10], RESUME examines the association of adverse and stressful experiences in childhood and adolescence with various outcomes in young adulthood including criminal behavior, aggressive behavior, mental and physical health, as well as social adjustment. The sample consists of 2,500 randomly selected individuals (1,314 females; 1,186 males) born between 1987 and 1991. At the time of study start, the participants were between 20 and 24 years old (mean age = 22.15, *SD* = 1.38). Participants were initially recruited from a pool of 20,827 individuals who had been drawn from a national population register at Statistics Sweden and who also had a valid phone number. To investigate whether non-participants (those who had declined, were never reached, or the target number of 2,500 had been reached) differed from participants, a comparison was made between 30 randomly selected non-participating males and females and the included 2,500 participants. No significant differences between participants and non-participants were found on key outcomes and demographics, with the exception of having ‘been forced to participate in sexual activities’, which was only reported by the participants [9].

The data were collected between March and December, 2011. Trained interviewers administered the study protocol, including informed consent, a face-to-face interview, questionnaires and collection of DNA saliva samples. The questionnaires were completed by the participants on an iPad. The entire session took on average 1.5 hour and the participants received a small monetary compensation for their participation. In the present study, only data from the questionnaire were used. For more details regarding study protocol and procedure, see [9] and regarding previous publication [10].

***Measures***

*The Alcohol Use Disorders Identification Test (AUDIT)*

All participants were asked to complete the 10-item AUDIT questionnaire regarding quantity (number of standard drinks = 12 g ethanol) and frequency of drinking as well as alcohol-related problems. The results for each question (score range 0–4) were summed to produce the AUDIT score (i.e. total score range 0–40). Scoring above eight points in males has been suggested as index of problematic drinking [11].

*Parent-Child Relationship.* Parent-child relationship was measured using the following items: “When you were growing up (0 - 18 years), how often did your (1) mother, (2) father, show clearly that they liked you, for example, by asking it or giving you a hug or kiss?” These items had a four-point response format, running from ”not very close at all” to ”very close”. Thereafter a parent-child relationship summation index (ranging from 0 to 6) was created with a higher score indicating a more positive parent-child relationship.

***Genotyping***

DNA was extracted from 200µl of saliva collected with the Oragene self-collection kit (DNA Genotek®) using the silica-based Kleargene DNA extraction method. Genotyping analyses of single nucleotide polymorphism (SNP) rs1360780 were performed using the Kbioscience Allele-Specific Polymorphism assay based on competitive allele-specific PCR and bi-allelic scoring of the single nucleotide polymorphism (SNP). No-template control samples were included to enable the detection of contamination or non-specific amplification.

**Table S1**

Primer pairs used to assess the gene expression with their respective annealing temperature

| **Gene** | **Gene name** | **Accession number (Genebank)** | **Primers** | **T (ᵒC)** | **Product length** |
| --- | --- | --- | --- | --- | --- |
| *Actb* | Actin, Beta | NM_031144.3 | Forward: 5’ CACTGCCGCATCCTCTTCCT 3’ | 60 | 81 |
|  |  |  | Reverse: 5’ AACCGCTCATTGCCGATAGTG 3’ |  |  |
| *Fkbp5* | FK506 Binding Protein 5 | NM_001012174.1 | Forward: 5’TGGTCTGACTCTCGTGTTTCTTG 3’ | 63 | 124 |
|  |  |  | Reverse: 5’CGCAGGGTGTACGCCAAC 3’ |  |  |
| *Gapdh* | Glyceraldehyde-3-Phosphate Dehydrogenase | NM_017008.4 | Forward: 5’ACATGCCGCCTGGAGAAACCT 3’ | 60 | 90 |
|  |  |  | Reverse: 5’GCCCAGGATGCCCTTTAGTGG 3’ |  |  |
| *Rpl19* | Ribosomal Protein L19 | [NM_031103.1](http://www.ncbi.nlm.nih.gov/entrez/viewer.fcgi?db=nucleotide&id=14389296) | Forward: 5’ TCGCCAATGCCAACTCTCGTC 3’ | 62 | 89 |
|  |  |  | Reverse: 5’ AGCCCGGGAATGGACAGTCAC 3’ |  |  |

**Table S2**

PCR efficiency and range of Cq values for *Fkbp5* expression

| **Region of interest** | **Mean PCR efficiency for *Fkbp5*** | **Cq value range** |
| --- | --- | --- |
| Amygdala | 1.896 | 24.47 – 28.47 |
| Cingulate cortex | 1.878 | 25.13 – 28.62 |
| Dorsal striatum | 1.886 | 24.89 – 29.22 |
| Medial prefrontal cortex | 1.887 | 25.74 – 27.64 |
| Nucleus accumbens | 1.895 | 26.16 – 32.44 |
| Ventral tegmental area | 1.893 | 27.24 – 30.93 |

**Table S3**

Group-wise bivariate correlations between expression of *Fkbp5* in different brain regions as well as with corticosterone levels. Non-significant correlations are not reported.

| **Group** | ***Fkbp5* relative expression** | | | ***r_s_*** |
| --- | --- | --- | --- | --- |
|  | Brain region | Brain region |  | |
| MS15 W | dStr | mPFC | 0.733^*^ | |
| MS360 W | Amy | mPFC | 0.648^*^ | |
| MS360 W | Amy | Acb | -0.673^*^ | |
| MS360 E | Amy | dStr | 0.480^*^ | |
| **MS360 E** | **Amy** | **mPFC** | **0.740**^*^ | |
| **MS360 E** | **CCx** | **mPFC** | **0.626**^*^ | |
| MS360 E | CCx | dStr | 0.462^*^ | |
| MS360 E | dStr | mPFC | 0.487^*^ | |
|  | Brain region | Hormone |  | |
| MS15 E | Amy | Corticosterone (ng/ml) | 0.886^*^ | |
| **MS15 E** | **mPFC** | **Corticosterone (ng/ml)** | **-0.943**^*^ | |
| MS360 E | Acb | Corticosterone (ng/ml) | -0.573^*^ | |

[bold]: significant after Bonferroni correction

Acb: nucleus accumbens; Amy: amygdala; CCx: cingulate cortex; dStr: dorsal striatum ; E: ethanol drinking; mPFC: medial prefrontal cortex; MS: maternal separation (15 or 360 min); *r_s_* : Spearmann’s correlation coefficient; S: single housed; VTA: ventral tegmental area; W: water drinking. ^*^: p < 0.005

**Table S4**

Demographics on the young adults of the RESUME cohort (N = 838)

| *FKBP5* genotype | CC (n = 446) | TC (n = 337) | TT (n = 55) |
| --- | --- | --- | --- |
|  | Mean ± SD (range) | | |
| Age, years | 22.1 ± 1.4 (20 – 24) | 22 ± 1.4 (20 – 24) | 22 ± 1.3 (20 – 24) |
| Age of first intoxication, years | 15.6 ± 1.9 (11- 22) | 15.8 ± 1.9 (11 - 22) | 15.9 ± 1.7 (12 - 19) |
| Alcohol consumed in a typical occasion, drinks | 5 – 6 | 5 – 6 | 5 – 6 |
| AUDIT score | 7.6 ± 5.1 (0 – 28) | 7.3 ± 5.1 (0 – 25) | 8.8 ± 5.9 (0 – 29) |
| Parent-child relationship (0 – 18 years) | 4.4 ± 1.5 (0 – 6) | 4.3 ± 1.5 (0 – 6) | 4.1 ± 1.5 (1 – 6) |

**Figure S1**


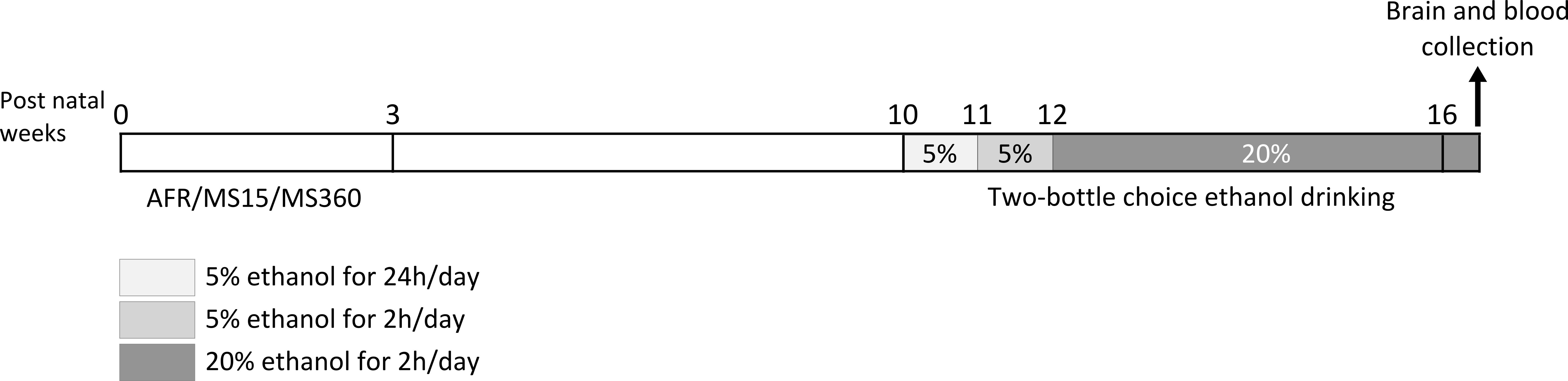


Study scheme of the maternal separation and free-choice voluntary ethanol drinking experimental model. MS: Maternal separation (15/360 min).

**Figure S2**

Gel images of *Fkbp5* primer specificity in the regions of interest.
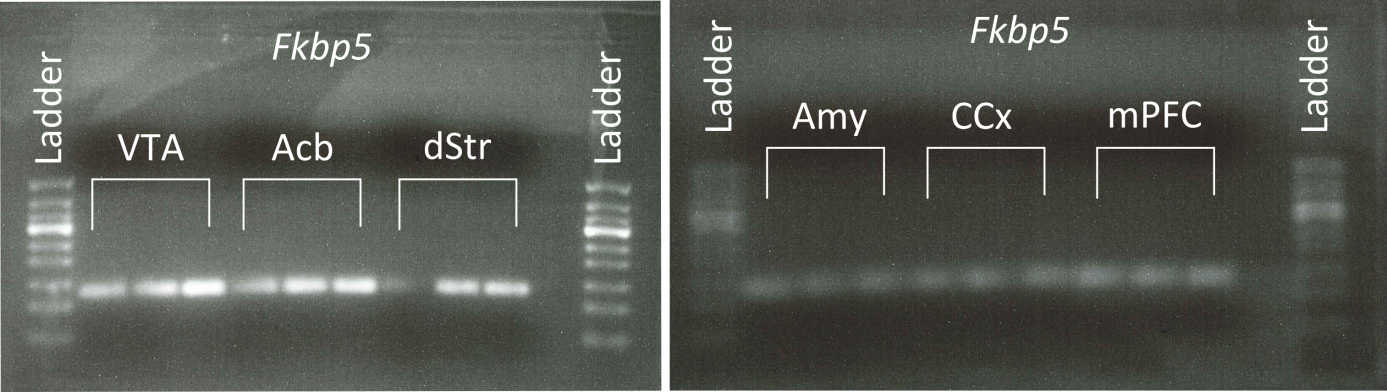
**Figure S3**

RNA integrity test images for all the regions studied


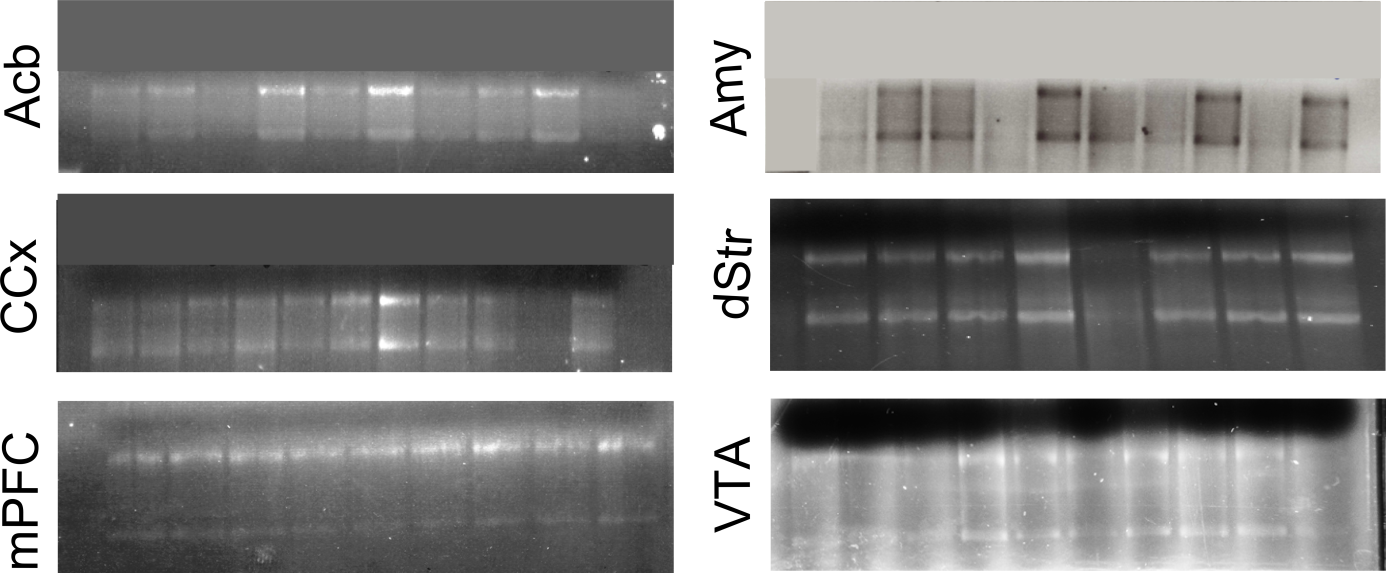


**Figure S4**

Interaction effects between maternal separation and ethanol drinking on *Fkbp5* expression in the ventral tegmental area and nucleus accumbens.

**
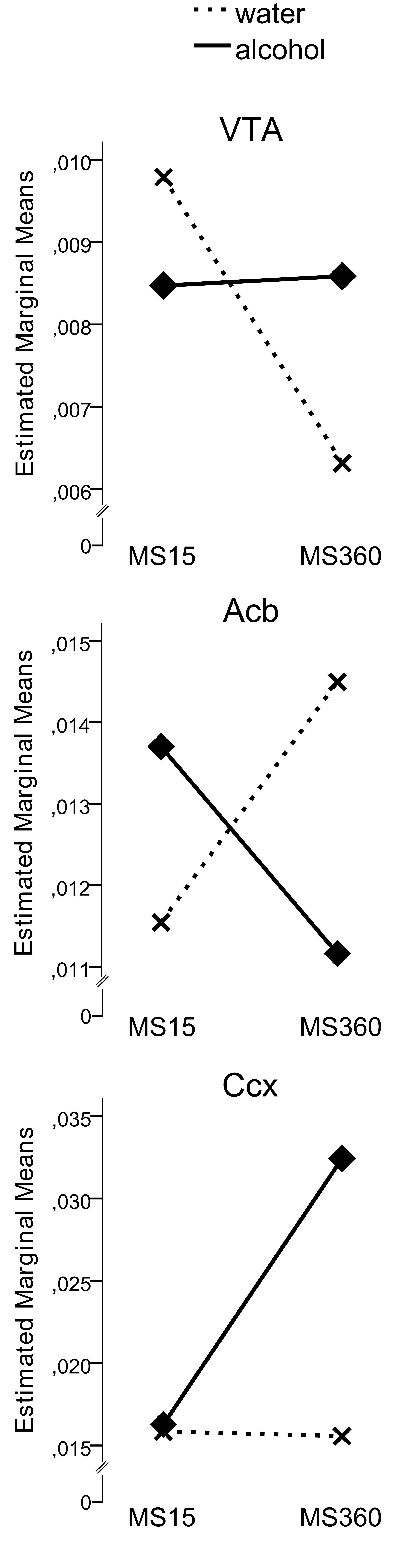
**

**REFERENCES**

1. Bendre M, Comasco E, Nylander I, Nilsson KW (2015) Effect of voluntary alcohol consumption on Maoa expression in the mesocorticolimbic brain of adult male rats previously exposed to prolonged maternal separation. Translational psychiatry 5:e690. doi:10.1038/tp.2015.186

2. Vrettou M, Granholm L, Todkar A, Nilsson KW, Wallen-Mackenzie A, Nylander I, Comasco E (2015) Ethanol affects limbic and striatal presynaptic glutamatergic and DNA methylation gene expression in outbred rats exposed to early-life stress. Addiction biology. doi:10.1111/adb.12331

3. Comasco E, Todkar A, Granholm L, Nilsson KW, Nylander I (2015) Alphalpha 2a-Adrenoceptor Gene Expression and Early Life Stress-Mediated Propensity to Alcohol Drinking in Outbred Rats. International journal of environmental research and public health 12 (7):7154-7171. doi:10.3390/ijerph120707154

4. Todkar A, Granholm L, Aljumah M, Nilsson KW, Comasco E, Nylander I (2015) HPA Axis Gene Expression and DNA Methylation Profiles in Rats Exposed to Early Life Stress, Adult Voluntary Ethanol Drinking and Single Housing. Frontiers in molecular neuroscience 8:90. doi:10.3389/fnmol.2015.00090

5. Nylander I, Roman E (2013) Is the rodent maternal separation model a valid and effective model for studies on the early-life impact on ethanol consumption? Psychopharmacology (Berl) 229 (4):555-569. doi:10.1007/s00213-013-3217-3

6. Gustafsson L, Nylander I (2006) Time-dependent alterations in ethanol intake in male wistar rats exposed to short and prolonged daily maternal separation in a 4-bottle free-choice paradigm. Alcohol Clin Exp Res 30 (12):2008-2016. doi:10.1111/j.1530-0277.2006.00247.x

7. Palm S, Nylander I (2014) Alcohol-induced changes in opioid peptide levels in adolescent rats are dependent on housing conditions. Alcohol Clin Exp Res 38 (12):2978-2987. doi:10.1111/acer.12586

8. Ruijter JM, Ramakers C, Hoogaars WMH, Karlen Y, Bakker O, van den Hoff MJB, Moorman AFM (2009) Amplification efficiency: linking baseline and bias in the analysis of quantitative PCR data. Nucleic Acids Res 37 (6):e45-e45. doi:10.1093/nar/gkp045

9. Cater Å, Andershed A-K, Andershed H (2014) Youth Victimization in Sweden: Prevalence, Characteristics and Relation to Mental Health and Behavioral Problems in Young Adulthood. Child Abuse & Neglect 38: 1290-1302

10. Tuvblad C, Narusyte J, Comasco E, Andershed H, Andershed AK, Colins OF, Fanti KA, Nilsson KW (2016) Physical and verbal aggressive behavior and COMT genotype: Sensitivity to the environment. American journal of medical genetics Part B, Neuropsychiatric genetics : the official publication of the International Society of Psychiatric Genetics. doi:10.1002/ajmg.b.32430

11. Babor TF, Higgins-Biddle JC, Saunders JB, Monteiro MG (2001) The Alcohol Use Disorders Identification Test - Guidelines for Use in Primary Care.
